# The Association Between Severity of Constipation and Oral Frailty Index-8 in the JUSTICE-TOKYO Study: A Cross-Sectional Study

**DOI:** 10.3390/biomedicines13040813

**Published:** 2025-03-28

**Authors:** Tsutomu Takeda, Daisuke Asaoka, Hiroyuki Kiko, Takuya Kanazawa, Osamu Nomura, Shotaro Oki, Mariko Hojo, Koji Sugano, Kei Matsuno, Hiroyuki Inoshita, Yuji Nishizaki, Naotake Yanagisawa, Mitsuyo Shinohara, Akihito Nagahara, Katsumi Miyauchi

**Affiliations:** 1 Department of Gastroenterology, Faculty of Medicine, Juntendo University, Tokyo 113-8421, Japan; t.kanazawa.hs@juntendo.ac.jp (T.K.); s-oki@juntendo.ac.jp (S.O.); mhojo@juntendo.ac.jp (M.H.); nagahara@juntendo.ac.jp (A.N.); 2 Department of Gastroenterology, Juntendo Tokyo Koto Geriatric Medical Center, Tokyo 136-0075, Japan; daisuke@juntendo.ac.jp (D.A.); h.kiko.yd@juntendo.ac.jp (H.K.); onomura@juntendo.ac.jp (O.N.); 3 Department of Respiratory Medicine, Juntendo Tokyo Koto Geriatric Medical Center, Tokyo 136-0075, Japan; ksugano@juntendo.ac.jp (K.S.); kmatsuno@juntendo.ac.jp (K.M.); 4 Department of Nephrology, Juntendo Tokyo Koto Geriatric Medical Center, Tokyo 136-0075, Japan; 5 Medical Technology Innovation Center, Juntendo University, Tokyo 113-8421, Japan; ynishiza@juntendo.ac.jp (Y.N.); n-yanagisawa@juntendo.ac.jp (N.Y.); 6 Department of Oral and Maxillofacial Surgery, Juntendo University Hospital, Tokyo 113-8421, Japan; mitsuyo@juntendo.ac.jp; 7 Department of Pathophysiological Research and Therapeutics for Gastrointestinal Diseases, Juntendo University, Tokyo 113-8421, Japan; 8 Department of Cardiology, Juntendo Tokyo Koto Geriatric Medical Center, Tokyo 136-0075, Japan; ktmmy@juntendo.ac.jp

**Keywords:** constipation scoring system, Oral Frailty Index-8, sarcopenia, Izumo scale

## Abstract

**Background/Objectives**: Reports on oral frailty as a risk factor for chronic constipation are scarce. In this study, we examined the relationship between Oral Frailty Index-8 (OFI-8) and constipation severity. **Methods**: This cross-sectional analysis involved patients aged ≥65 years (outpatients between November 2020 and November 2021). Patient background (age, sex, body mass index, medical history, lifestyle history, and oral medications), a constipation severity questionnaire (Constipation Scoring System [CSS]), grip strength, walking speed, skeletal muscle mass index (dual-energy X-ray absorptiometry), a frailty questionnaire, an oral frailty questionnaire (OFI-8), an abdominal symptoms quality of life (QOL) questionnaire (Izumo scale), a swallowing evaluation questionnaire (10-item Eating Assessment Tool [EAT-10]), a chronic obstructive pulmonary disease (COPD) evaluation questionnaire (COPD assessment test [CAT]), a simplified QOL evaluation (EuroQol-five dimensions [EQ-5D]), the Dietary Variety Score, a nutritional evaluation (CONtrolling NUTritional Status [CONUT] score), and the 15-item Geriatric Depression Scale (GDS-15) were analyzed. Risk factors for constipation severity (CSS) were examined using multivariate analysis. Patients with advanced gastrointestinal cancer, inflammatory bowel disease, and active gastroduodenal ulcer were excluded. **Results**: In total, 1029 patients (male/female: 450/579; mean age: 78.3 ± 6.1 years; mean body mass index: 22.9 ± 3) were included. Multivariate analysis demonstrated a significant association between CSS and OFI-8 (β = 0.065), EAT-10 (β = 0.061), sarcopenia (β = 0.050), laxative (β = 0.126), constipation-related QOL score (β = 0.625), diarrhea-related QOL score (β = −0.064), and CAT (β = 0.061). **Conclusions**: Comprehensive risk factors associated with CSS included a high oral frailty score, impaired swallowing (EAT-10), sarcopenia, laxative use, a high constipation QOL score, a low diarrhea QOL score, and COPD assessment through CAT.

## 1. Introduction

As the population ages, chronic constipation is becoming a disease that hinders healthy longevity. The prevalence of chronic constipation was 14% (range: 2–35%) in a meta-analysis [1], and the incidence rate increases with age [2,3]. Chronic constipation also reduces quality of life (QOL) [4,5,6]. Furthermore, a link with systemic diseases, including cardiovascular disease, has been suggested [7,8]. The number of patients with chronic constipation will increase in the future, and these patients have been associated with a poorer prognosis [9,10]. Moreover, a greater financial burden on medical systems is anticipated [11], emphasizing the need for appropriate treatment.

In today’s aging society, caring for elderly individuals who are bedridden and require assistance is becoming a challenge. Intervention for frailty and sarcopenia to prevent bedriddenness and the need for nursing care is essential. Frailty places elderly individuals at high risk for adverse health outcomes [12,13]. Recently, the revised Japanese version of the Cardiovascular Health Study criteria (revised J-CHS criteria) was published [14]. Within this framework, a new concept called “oral frailty” was developed, focusing on mild decline in oral function. It has become clear that oral frailty appears at a preliminary stage of frailty, and has a significant impact on the progression of frailty [15]. When oral function declines, dietary imbalance occurs, nutritional balance is disrupted, and the risk of falling into a state of low nutrition and requiring nursing care increases. Thus, to achieve healthy longevity, diagnosis of oral frailty, frailty, and sarcopenia and appropriate intervention are important. Furthermore, oral frailty leads to restricted food intake and indigestion, which may contribute to constipation. The oral intake and digestion of nutrients play a crucial role in causing undernutrition in the elderly. However, the relationship between constipation severity and oral frailty in older individuals has not been thoroughly examined.

To date, few reports have evaluated oral frailty and sarcopenia as risk factors for chronic constipation. A previous cohort study of 1042 patient who visited a geriatric hospital revealed high rates of sarcopenia, frailty, and pre-frailty (21.4%, 16.5%, and 51.9%, respectively) [16].

In the present analysis, we investigated, in a multidisciplinary setting, the relationship between constipation severity and oral frailty, including sarcopenia, among a large number of elderly outpatients at an institution specializing in geriatric medicine.

## 2. Materials and Methods

### 2.1. Study Cohort

This single-center, cross-sectional analysis was conducted using baseline data obtained from the prospective cohort JUSTICE-TOKYO (JUntendo Sarcopenia regisTratIon of exploring for prediCtors and prognosis in Elderly in TOKYO) study [16]. The study involved consecutive elderly outpatients (age: ≥65 years) who visited the Juntendo Tokyo Koto Geriatric Medical Center between November 2020 and November 2021. The investigation included a 4-year follow-up, as well as annual assessments of survival, incidence of falls, hospitalization, and skeletal muscle mass. Baseline data were recorded at the time of enrollment and entered into the Research Electronic Data Capture (REDCap) system [17].

### 2.2. Inclusion Criteria

Outpatients aged ≥ 65 years attending Juntendo Tokyo Koto Geriatric Medical Center were eligible for enrollment in the JUSTICE-TOKYO study. Consecutive patients in the stated period were included in the study based on obtaining the following complete information from personal health records: (i) personal characteristics (age, gender, and body mass index), (ii) constipation scoring system, (iii) Oral Frailty Index-8, (iv) dual-energy X-ray absorptiometry, walking speed, and handgrip strength.

### 2.3. Exclusion Criteria

Patients were excluded for the following reasons: (i) inability to walk independently because of severe osteoarthritis or neuromuscular disease; (ii) immobility; (iii) delirium tremens at presentation; (iv) a history of advanced gastrointestinal cancer, inflammatory bowel disease, or an active gastroduodenal ulcer, as well as acute gastrointestinal, renal, cerebrovascular, coronary, hepatic, and respiratory events; (v) an inability to be interviewed by questionnaire; (vi) administration of enteral nutrition formulas; and (vii) a predicted life expectancy of <1 year because of malignant disease.

### 2.4. Measurement of Baseline Variables

Information regarding the measurement of baseline variables is provided in Table 1 [14,18,19,20,21,22,23,24,25,26,27,28,29]. Patient data were collected within 3 months following enrollment.

### 2.5. Endpoint

The Constipation Scoring System (CSS) was used as the dependent variable (endpoint) to examine factors influencing constipation severity.

### 2.6. Statistical Analysis

We assessed the risk factors for CSS by univariate analysis (UVA) and multivariate analysis (MVA). In the UVA, correlations between CSS and clinical parameters were calculated using Pearson’s correlation coefficients (r) for quantitative variables and correlation ratios (η) for nominal scales. In the multiple regression analysis (MRA), the CSS was employed as a dependent variable. Significant independent variables (*p*-value < 0.2) in the UVA were included in the MVA. Age, QOL (EuroQol-five dimensions [EQ-5D]), history of falls, day care use, handgrip strength, walking speed, comorbidities (cerebral infarction/hemorrhage, chronic hepatic disease, hypertension, atrial fibrillation, frailty, sarcopenia), use of agents (i.e., acid secretion suppressants, laxatives, and number of oral medications), oral function (Oral Frailty Index-8 (OFI-8), the 10-item Eating Assessment Tool (EAT-10), the Mini-Mental State Examination (MMSE), the 15-item Geriatric Depression Scale (GDS-15), the chronic obstructive pulmonary disease (COPD) assessment test (CAT), hypozincemia, the CONtrolling NUTritional Status (CONUT) score, the Dietary Variety Score (DVS), and QOL scores related to reflux, upper abdominal pain, fullness, constipation, and diarrhea were considered as independent variables. Risk factors for CSS were subjected to MRA via a stepwise method. A variance inflation factor ≥10 denoted multicollinearity. Pairwise deletion was applied in MRA to handle missing data and enhance statistical power. The SPSS version 28 software was used for all statistical analyses (IBM Corporation, Armonk, NY, USA). *p*-values < 0.05 denoted statistically significant differences.

## 3. Results

### 3.1. Clinical Characteristics

**Figure 1:**
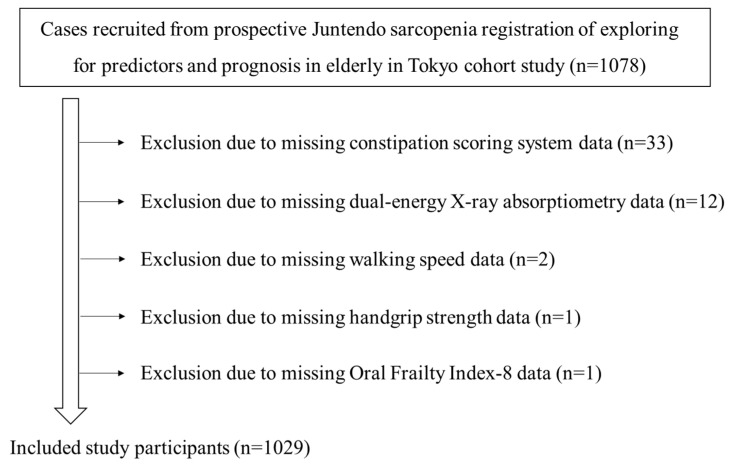
Flow diagram of study participants.

The clinical characteristics of the 1029 subjects (male: *n* = 450; female: *n* = 579; mean age: 78.3 ± 6.1 years; mean body mass index: 22.9 ± 3.9 kg/m^2^) are shown in Table 2. The participant recruitment flow diagram is shown in Figure 1. Among the eligible subjects, 562 and 226 were users of acid secretion suppressants and laxatives, respectively. The mean score of the CSS was 3.4.

### 3.2. Correlation Between CSS and Various Clinical Parameters (UVA)

Table 3 shows the correlation between CSS and variables in the UVA. Significant differences were recorded in age, QOL (EQ-5D), history of falls, day care use, frailty, handgrip strength, walking speed, sarcopenia, laxatives, number of oral medications, OFI-8, EAT-10, MMSE, GDS-15, QOL scores related to reflux, upper abdominal pain, fullness, constipation, and diarrhea, CAT, CONUT score, and DVS.

### 3.3. Association Between CSS and Other Variables (MRA)

Based on the MRA, the variables of OFI-8, EAT-10, sarcopenia, laxative use, the QOL score related to constipation, the QOL score related to diarrhea, and the CAT were linked to the CSS (Table 4).

## 4. Discussion

Herein, we present the first large cross-sectional analysis investigating risk factors associated with constipation severity (including oral frailty) in elderly outpatients. Our results clarified that OFI-8, EAT-10, sarcopenia, laxative use, QOL scores related to constipation and diarrhea, and the CAT were related to the CSS. Elderly patients with constipation may also have oral frailty, swallowing dysfunction, sarcopenia, or COPD. The relationship between constipation and oral frailty, sarcopenia, or COPD in elderly individuals may apply to diagnosis and treatment. In the future, early intervention for these conditions may be beneficial for the clinical management of constipation.

In our study, OFI-8 and EAT-10, as measures of oral function, were associated with the CSS. Oral frailty significantly impacts the progression of frailty [15], and is correlated with physical frailty [30]. It has become clear that a deterioration in the oral environment, decreased bite force and tongue pressure, a reduced number of teeth, and decreased saliva secretion have a significant impact on the progression of frailty and sarcopenia. The progression of oral frailty may reduce mechanical digestion through chewing and stirring, as well as chemical digestion, and may affect digestive and absorption disorders. We previously reported that frailty is associated with CSS scores [31]. Oral frailty has been identified as a precursor to frailty. Moreover, impaired oral and swallowing function can cause indigestion and reduced nutritional status. These observations suggest that this may be associated with frailty as well as constipation. In this study, the GDS-15 was significantly correlated with the CSS, suggesting that various factors, such as depressive symptoms from a psychological perspective, and living alone or economic hardship from a social perspective, may affect constipation. Lin et al. reported that depression was associated with oral frailty and physical frailty [32]. Therefore, when treating constipation in elderly individuals with oral frailty and frailty, comprehensive multidisciplinary support is required. The present findings indicate that improving oral frailty may lead to improved constipation, and prompt diagnosis of oral frailty is essential.

Sarcopenia was also significantly associated with the CSS. In previous research, we demonstrated that sarcopenia was associated with the CSS retrospectively [33]. Of 310 outpatients aged ≥65 years, 83 (26.8%) had sarcopenia, and the CSS score was significantly higher in the group with sarcopenia versus that without (4.9 ± 4.9 vs. 3.6 ± 3.6, respectively, *p* = 0.009). MRA revealed that the CSS score was significantly associated with sarcopenia, the Izumo scale QOL score related to constipation, the use of laxatives and intestinal motility regulators, and the Bristol Stool Form Scale (BSFS) score. The results of this study support this evidence. In a cross-sectional study, Park et al. assessed the relationship between constipation and sarcopenia in 1278 community-dwelling elderly individuals [34]. Logistic regression analysis demonstrated that the incidence of functional constipation was significantly higher in the group with sarcopenia versus that without (odds ratio: 2.02; 95% confidence interval: 1.34–3.04). An association between low physical activity and chronic constipation has also been reported in other studies [35]. It has been shown that constipation is related to sarcopenia and, in geriatric care, it is necessary to understand and improve constipation, as well as the overall condition of the body, including the nutritional status and muscle weakness. In elderly individuals with sarcopenia, the severity of constipation is thought to increase due to various factors, including decreased appetite, decreased physical activity, decreased intestinal function, and the effects of polypharmacy [2,36]. Decreased function of the pelvic floor muscles is a cause of functional fecal evacuation disorders [37]. Furthermore, weakened abdominal muscles suppress the increase in intra-abdominal pressure that accompanies straining. Therefore, it is important to prevent muscle weakness throughout the body, including improving the function of the abdominal and pelvic floor muscles.

Moreover, our study showed that the CSS was correlated with abdominal symptoms. The data demonstrated that the QOL scores related to constipation and laxative use were positively associated, whereas the QOL score related to diarrhea was negatively associated with CSS. We previously reported that the CSS was associated with the QOL score related to constipation and the BSFS score [33]. The feeling of incomplete evacuation or hard stools that continue for days could affect QOL, while constipation-related symptoms may include reduced appetite and limited activity in the elderly. The CSS was also associated with laxative use. Yamamoto et al. reported that individuals who had hard stools, used multiple laxatives, or spent more money on treatment had low QOL scores [38]. High constipation severity may lead to increased use of laxatives, which might affect QOL. The QOL score related to diarrhea was positively correlated, but negatively associated with CSS. This finding may be attributed to laxative use by many patients and high diarrhea scores in some patients, despite having constipation. Based on previous reports, low BSFS scores are associated with CSS [33]; in an MVA including various confounding factors, QOL scores related to diarrhea were thought to be negatively associated with CSS.

Regarding pulmonary function, the CAT was associated with CSS. In a systematic review and meta-analysis, 15.5–34% of patients with COPD had sarcopenia [39]. In addition, COPD was correlated or associated with constipation symptoms [40,41]. Kagiali et al. revealed that CAT scores were higher in frail patients versus non-frail patients [42]. In our opinion, COPD-related decreased activity and muscle weakness might influence the severity of constipation.

In a systematic review and meta-analysis of data on chronic idiopathic constipation in the community, female sex and age were identified as risk factors for constipation [1]. This review focused on individuals aged ≥15 years, reporting that the risk of constipation increased with age. In contrast, the present analysis did not detect a significant association between sex and constipation. This discrepancy may be caused by differences in the characteristics of our study population, which primarily comprised elderly individuals (mean age: 78.3 years), and the fact that the research was conducted in a university hospital specializing in geriatric medicine. In studies involving a broader general population, including younger adults, age might emerge as a more prominent risk factor. Additionally, while sex has been identified as a risk factor in the general population, with females being more susceptible, our findings indicated no significant association between sex and constipation risk. According to an overview of the 2022 Basic Survey on National Living Conditions by the Ministry of Health, Labor and Welfare in Japan [43], the prevalence of self-reported constipation was 27.5% and 43.7% among males and females, respectively (68.1% and 73.8% for those aged ≥65 years, respectively). While females generally exhibited higher rates of self-reported constipation, the sex disparity narrowed with advancing age, as the prevalence among males increased significantly in the older population. This trend suggests that the lack of sex association observed in this study may be attributed to the age distribution of the population, predominantly comprising elderly individuals.

The current analysis had several limitations. Firstly, the study subjects were limited to outpatients from a single institution specializing in geriatric medicine. Secondly, we did not take into account other background variables, such as exercise routines, dietary patterns, occupations, education level, and marital status. This limits the generalizability of the findings to older individuals; moreover, the results may have been overestimated due to unhealthy subject bias. Thirdly, this study did not evaluate oral dysfunction or oral bacteria. In the future, collaboration with dentists will be necessary in order to examine oral function.

## 5. Conclusions

In conclusion, this study revealed that a high oral frailty score, impaired swallowing (EAT-10), sarcopenia, laxative use, a high QOL score related to constipation, a low QOL score related to diarrhea, and COPD assessment (CAT) were associated with CSS. Intervention studies are warranted to assess the effect of improvements in these factors on chronic constipation.

## Figures and Tables

**Table 1:** Measurement of baseline variables.

**Patient profile**
Age (years)
Sex (male/female)
Body mass index (kg/m^2^)
Brinkman Index: the number of cigarettes smoked per day multiplied by the number of years of smoking
Alcohol drinking habits: 0, 1, and 2 indicate rarely, 1–4 days per week, and 5–7 days per week, respectively
QOL (EQ-5D): 0 and 100 indicate worst and perfect health, respectively
History of falls
History of day care use
SMI (kg/m^2^), measured using DXA scanning (GE Healthcare, Madison, WI, USA)
Handgrip strength (kg)
Walking speed (m/s)
**Comorbidities**
Cerebral infarction/hemorrhage
Myocardial infarction
Hospitalization for heart failure
Interstitial pneumonia
Chronic hepatic disease
History of malignant disease
Hypertension
Diabetes mellitus
Atrial fibrillation
Osteoporosis: diagnosed according to the criteria of the Japanese Society for Bone and Mineral Research
Frailty: diagnosed using the J-CHS criteria, adapted from the original Cardiovascular Health Study criteria
Sarcopenia: defined using the diagnostic algorithm recommended by the Asian Working Group for Sarcopenia: 2019 Consensus Update on Sarcopenia Diagnosis and Treatment
**Use of therapeutic agents**
Statins
Acid secretion suppressants
Laxatives
Non-steroidal anti-inflammatory drugs
Number of oral medicines
**Oral function**
Oral frailty: evaluated based on the Oral Frailty Index-8 (OFI-8) score using an 8-point scale
Dysphagia: assessed based on the 10-item Eating Assessment Tool (EAT-10)
**Severity of constipation**
CSS score (frequency of bowel movements, painful evacuation, incomplete evacuation, abdominal pain, length of time per attempt, assistance for evacuation, unsuccessful attempts at evacuation per 24 h, and duration of constipation). The overall CSS score (i.e., the sum of the point scores for each item) ranged from 0–30, with higher scores indicating worse constipation symptoms.
**Neuropsychological examination**
Mini-Mental State Examination (MMSE)
15-item Geriatric Depression Scale (GDS-15)
**Abdominal symptom-related QOL scores**
This self-administered questionnaire (the Izumo scale) includes 15 items in five domains (i.e., reflux, upper abdominal pain, fullness, constipation, and diarrhea). Each item is scored using a Likert scale (range: 0–5). Domain-specific QOL impairment on the Izumo scale is ranked from 0 (indicating no QOL impairment) to 15 (indicating severe QOL impairment).
**Pulmonary function**
SpO_2_ (%)
The impact of COPD on the health status was evaluated based on the CAT, consisting of eight items. A CAT score ≥10 indicates severe symptoms.
**Nutritional status**
Hypozincemia was defined as treatment with zinc acetate hydrate (Novelzin^®^ Tablets; Nobelpharma K.K., Tokyo, Japan) or serum zinc levels < 80 µg/dL.
The CONUT score (range: 0–12) was used to investigate the objective nutritional status. This score was calculated using the serum albumin levels, total cholesterol levels, and total lymphocyte count.
The DVS comprises 10 food-based components.

CAT, chronic obstructive pulmonary disease assessment test; CSS, Constipation Scoring System; CONUT, CONtrolling NUTritional status; DVS, Dietary Variety Score; DXA, dual-energy X-ray absorptiometry; EAT-10, 10-item Eating Assessment Tool; EQ-5D, EuroQol-five dimensions; GDS-15, 15-item Geriatric Depression Scale; J-CHS, the Japanese version of the Cardiovascular Health Study; MMSE, Mini Mental State Examination; SMI, skeletal muscle mass index; QOL, quality of life; SpO_2_, arterial oxygen saturation.

**Table 2:** Clinical characteristics of study patients (*n* = 1029).

**Patient profile**	
Mean age (years)	78.3 ± 6.1 **
Sex	
Male	450 (43.7) *
Female	579 (56.3) *
Body mass index (kg/m^2^)	22.9 ± 3.9 **
Brinkman Index	355.9 ± 615.1 **
Alcohol drinking habit	0.5 ± 0.8 **
QOL (EQ-5D)	74.9 ± 17.0 **
History of falls	207 (20.1) *
History of day care use	94 (9.1) *
SMI	6.3 ± 0.9 **
Handgrip strength (kg)	25.9 ± 8.5 **
Walking speed (m/s)	1.2 ± 0.4 **
**Comorbidities**	
Cerebral infarction/hemorrhage	80 (7.8) *
Myocardial infarction	47 (4.6) *
Hospitalization for heart failure	42 (4.1) *
Interstitial pneumonia	55 (5.3) *
Chronic hepatic disease	126 (12.2) *
History of malignant disease	55 (5.3) *
Hypertension	606 (58.9) *
Diabetes mellitus	319 (31.0) *
Atrial fibrillation	88 (8.6) *
Osteoporosis	337 (32.8) *
Frailty	174 (16.9) *
Sarcopenia	223 (21.7) *
**Use of therapeutic agents**	
Statins	430 (41.8) *
Acid secretion suppressants	562 (54.6) *
Laxatives	226 (22.0) *
Non-steroidal anti-inflammatory drugs	76 (7.4) *
Number of oral medicines	6.1 ± 3.5 **
**Oral function**	
Oral Frailty Index-8	3.9 ± 2.4 **
EAT-10	1.6 ± 3.6 **
**Severity of constipation**	
CSS score	3.4 ± 3.7 **
**Neuropsychological examination**	
MMSE	26.5 ± 3.1 **
GDS-15	4.2 ± 3.0 **
**Abdominal symptom-related QOL scores**	
Reflux	1.8 ± 2.4 **
Upper abdominal pain	1.1 ± 2.0 **
Fullness	1.6 ± 2.4 **
Constipation	2.2 ± 2.6 **
Diarrhea	2.1 ± 2.6 **
**Pulmonary function**	
SpO_2_ (%)	97.2 ± 2.4 **
CAT	8.6 ± 6.6 **
**Nutritional status**	
Hypozincemia	807 (78.4) *
CONUT score	1.0 ± 1.2 **
DVS	3.7 ± 2.2 **

Data are presented as * number (%) or ** mean ± standard deviation. CAT, chronic obstructive pulmonary disease assessment test; CSS, constipation scoring system; CONUT, CONtrolling NUTritional status; DVS, Dietary Variety Score; EAT-10, 10-item Eating Assessment Tool; EQ-5D, EuroQol-five dimensions; GDS-15, 15-item Geriatric Depression Scale; MMSE, Mini Mental State Examination; SMI, skeletal muscle mass index; QOL, quality of life; SpO_2_, arterial oxygen saturation.

**Table 3:** Correlation between constipation scoring system score and various clinical parameters.

**Patient profile**	**r, η**	***p*-Value**
Age (years)	0.083	0.007 ^†^
Sex (female)	0.008	0.792
Body mass index (kg/m^2^)	−0.033	0.291
Brinkman Index	0.016	0.673
Alcohol drinking habit	−0.036	0.246
QOL (EQ-5D)	−0.251	<0.001 ^†^
History of falls	0.166	<0.001 ^†^
History of day care use	0.143	<0.001 ^†^
SMI	−0.037	0.236
Handgrip strength (kg)	−0.086	0.006 ^†^
Walking speed (m/s)	−0.154	<0.001 ^†^
**Comorbidities**		
Cerebral infarction/hemorrhage	0.055	0.077
Myocardial infarction	0.000	0.987
Hospitalization for heart failure	0.003	0.923
Interstitial pneumonia	0.032	0.312
Chronic hepatic disease	0.045	0.147
History of malignant disease	0.030	0.336
Hypertension	0.052	0.094
Diabetes mellitus	0.011	0.718
Atrial fibrillation	0.049	0.114
Osteoporosis	0.013	0.667
Frailty	0.193	<0.001 ^†^
Sarcopenia	0.075	0.017 ^†^
**Use of therapeutic agents**		
Statins	0.009	0.761
Acid secretion suppressants	0.054	0.086
Laxatives	0.314	<0.001 ^†^
Non-steroidal anti-inflammatory drugs	0.001	0.975
Number of oral medicines	0.153	<0.001 ^†^
**Oral function**		
Oral Frailty Index-8	0.294	<0.001 ^†^
EAT-10	0.291	<0.001 ^†^
**Neuropsychological examination**		
MMSE	−0.063	0.049 ^†^
GDS-15	0.285	<0.001 ^†^
**Abdominal symptom-related QOL scores**		
Reflux	0.285	<0.001 ^†^
Upper abdominal pain	0.279	<0.001 ^†^
Fullness	0.337	<0.001 ^†^
Constipation	0.694	<0.001 ^†^
Diarrhea	0.307	<0.001 ^†^
**Pulmonary function**		
SpO_2_ (%)	−0.014	0.645
CAT	0.329	<0.001 ^†^
**Nutritional status**		
Hypozincemia	0.054	0.085
CONUT score	0.282	<0.001 ^†^
DVS	−0.124	<0.001 ^†^

^†^ *p* < 0.05, significant difference; CAT, chronic obstructive pulmonary disease assessment test; CONUT, CONtrolling NUTritional status; DVS, Dietary Variety Score; EAT-10, 10-item Eating Assessment Tool; EQ-5D, EuroQol-five dimensions; GDS-15, 15-item Geriatric Depression Scale; MMSE, Mini Mental State Examination; SMI, skeletal muscle mass index; QOL, quality of life; SpO_2_, arterial oxygen saturation.

**Table 4:** Association between constipation scoring system score and other variables in multiple regression analysis.

**Covariates**	**β**	**t**	**95% CI**	***p*-Value**
Oral Frailty Index-8	0.065	2.461	0.020, 0.181	0.014 ^†^
EAT-10	0.061	2.294	0.009, 0.112	0.022 ^†^
Sarcopenia	0.050	2.176	0.045, 0.866	0.030 ^†^
Laxative	0.126	5.246	0.711, 1.561	<0.001 ^†^
QOL score related to constipation	0.625	22.613	0.830, 0.9987	<0.001 ^†^
QOL score related to diarrhea	−0.064	−2.404	−0.165, −0.017	0.016 ^†^
CAT	0.061	2.217	0.004, 0.064	0.027 ^†^

^†^ *p* < 0.05, significant difference; CAT, chronic obstructive pulmonary disease assessment test; CI, confidence interval; EAT-10, 10-item Eating Assessment Tool; QOL, quality of life.

## Data Availability

The data presented in this study are available on request from the corresponding author. The data are not publicly available, due to privacy and ethical restrictions.
